# Unsupervised clustering of electroclinical features in temporal lobe epilepsy: A data‐driven approach

**DOI:** 10.1002/epd2.70283

**Published:** 2026-05-19

**Authors:** Maria Vlachou, Bernadett Molnár, Helene Kaas, Jesper Tveit, Harald Aurlien, Martin Ejler Fabricius, Guido Rubboli, Lars Hageman Pinborg, Sándor Beniczky

**Affiliations:** ^1^ Department of Clinical Neurophysiology Danish Epilepsy Center Dianalund Denmark; ^2^ Neurobiology Research Unit and Epilepsy Clinic, Department of Neurology Copenhagen University Hospital Rigshospitalet Copenhagen Denmark; ^3^ Department of Clinical Medicine University of Copenhagen Copenhagen Denmark; ^4^ Natus Neuro Bergen Norway; ^5^ Department of Clinical Neurophysiology Haukeland University Hospital Bergen Norway; ^6^ Department of Clinical Neurophysiology Copenhagen University Hospital Rigshospitalet Copenhagen Denmark; ^7^ Department of Clinical Medicine, Danish Epilepsy Center Filadelfia University of Copenhagen Copenhagen Denmark; ^8^ Department of Clinical Neurophysiology Danish Epilepsy Centre Dianalund Denmark; ^9^ Copenhagen University Hospital Rigshospitalet Copenhagen Denmark

**Keywords:** epileptogenic zone, focal epilepsy, ictal EEG, seizure semiology, unsupervised clustering

## Abstract

**Objective:**

To identify clinically meaningful patterns in ictal electroclinical features of focal epilepsy using a data‐driven, unsupervised learning approach, and to assess whether such patterns can localize and lateralize the epileptogenic zone (EZ) more accurately than conventional electroclinical interpretation.

**Methods:**

Ictal scalp EEG and seizure semiology were systematically extracted using the SCORE system in 116 patients with drug‐resistant focal epilepsy who subsequently underwent resective surgery. Unsupervised clustering was applied separately to EEG and semiology features using k‐means and agglomerative hierarchical clustering. Cluster quality was evaluated using the silhouette score, within‐cluster sum of squares (WSS), between‐cluster sum of squares (BSS), the ratio of between‐cluster to within‐cluster variance (BSS/WSS), the proportion of variance explained by clustering (BSS/TSS), and the Calinski–Harabasz index. The ability of the resulting clusters to localize and lateralize the temporal lobe EZ was assessed and compared with the conventional approach reflecting current clinical practice, based on previously published lateralizing signs, semiology localizing to the temporal lobe, and scalp EEG localization of ictal onset.

**Results:**

Unsupervised clustering achieved strong structure for EEG and moderate for semiology data. K‐means clustering of EEG features yielded a silhouette score of .63, while hierarchical clustering of semiology features achieved a silhouette score of .54. Semiology‐based clusters showed substantial overlap with respect to both localization and lateralization. EEG‐based clusters localized and lateralized the temporal lobe EZ with an accuracy of 65.3% (95% CI: 55.0%–74.6%). This performance was comparable to conventional electroclinical interpretation using scalp EEG ictal onset (68.1%, 95% CI: 57.5%–77.5%). Interpretation based on seizure semiology achieved an accuracy of 71.3% (95% CI: 61.0–80.1%).

**Significance:**

Unsupervised clustering of ictal electroclinical features did not outperform conventional expert interpretation in localizing or lateralizing the EZ. These findings suggest that the limitations may be inherent to the observable ictal phenomena captured by scalp EEG and semiology. Future research should focus on identifying novel or complementary biomarkers to improve precision in epilepsy surgery evaluation.

**Key points**

- Unsupervised clustering was applied to ictal EEG and seizure semiology in focal epilepsy patients.
- EEG clustering showed strong structure and localized temporal EZ with 65% accuracy.
- Semiology‐based clusters could not localize temporal EZ while conventional interpretation achieved an accuracy of 71%.
- Clustering performance was comparable to scalp EEG but inferior to expert semiology analysis.
- Limitations appear intrinsic to ictal features, highlighting the need for novel biomarkers.

## 1 INTRODUCTION

Although epilepsy surgery is an effective and safe treatment for selected patients with focal drug‐resistant epilepsy (DRE), it remains underutilized and imperfect, emphasizing the need for improving presurgical evaluation.^1^, ^2^ Identification of the epileptogenic zone (EZ), the cortical area that must be removed to render the patient seizure‐free, is complex and challenging.^3^ The large volume of electroclinical data acquired from long‐term video‐EEG monitoring (LTM), the variability in seizure presentations, inter‐rater variability in feature extraction, and inconsistencies in free‐text reporting contribute to difficulties in interpretation.^4^

Standardized Computer‐based Organized Reporting of EEG (SCORE) is a structured common data element system, endorsed by the International Federation of Clinical Neurophysiology (IFCN) and International League Against Epilepsy (ILAE).^4^, ^5^ It has been implemented into a software package (SCORE‐EEG) for assisting clinicians to extract, document and report EEG and semiology features in a standardized way, using the unified terminology.^4^, ^5^, ^6^, ^7^ Previous studies indicated that using SCORE improves the quality of clinical interpretation and provides standardized datasets for research.^4^, ^5^, ^6^ The structured dataset enabled training of artificial intelligence (AI) models for automated and semiautomated analysis of clinical EEG.^8^, ^9^

AI methods have gained increasing attention in epileptology and have demonstrated utility in seizure detection, imaging analysis, and prediction of medical and surgical outcomes.^10^, ^11^ Machine learning approaches have been applied mainly to automated EEG signal analysis, including seizure detection, classification, and prediction.^12^, ^13^, ^14^ In contrast, studies based on clinical data more often rely on supervised learning to predict diagnosis or outcome rather than unsupervised techniques to identify data‐driven patient subgroups.^15^, ^16^

Although seizure semiology is central to clinical evaluation, data‐driven analyses of semiological features are relatively scarce; cluster analyses have been reported mainly in specific contexts such as psychogenic nonepileptic seizures and absence epilepsy, identifying distinct behavioral subtypes.^17^, ^18^ Recently, a study applied data‐driven clustering to systematically annotated electroclinical seizure data, demonstrating the feasibility of integrating semiology with EEG features to identify reproducible seizure subtypes.^19^ Most existing studies rely on single modalities or nonstandardized clinical descriptions, limiting comparability and reproducibility. While SCORE provides structured terminology for EEG reporting,^4^ clustering approaches using standardized electroclinical data—particularly systematically annotated seizure semiology, alone or combined with EEG features—remain largely unexplored.

Ictal EEG and semiology, recorded during LTM, are essential in the multimodal presurgical evaluation. Most studies evaluating their diagnostic value have been hypothesis‐driven and influenced by previous knowledge accumulated in the field. While intellectually rewarding, this approach bears intrinsic limitations, as it is potentially biased by a priori knowledge. To circumvent this, we used unsupervised machine learning methods to identify ictal EEG and semiology patterns that localize and lateralize the EZ. More specifically, we used unsupervised clustering of ictal EEG and semiological features in the SCORE database and assessed whether they can distinguish between temporal and extratemporal foci, and to lateralize the temporal foci better than the conventional clinical interpretation.

## 2 METHODS

### 2.1 Patient selection

Patients were identified from a cohort undergoing LTM at the Danish Epilepsy Center, Dianalund, Denmark, between 2006 and 2019. Inclusion criteria were^1^: history of focal DRE,^2^ resective epilepsy surgery,^3^ availability of LTM data (video and EEG), and^4^ documented surgical outcome at 12 months postoperatively. Patients who underwent hemispherectomy or callosotomy were excluded due to the distinct nature of these interventions. Of 167 patients initially identified, 116 consecutive patients fulfilled inclusion criteria and were analyzed.

### 2.2 Data collection and feature extraction

All ictal events were independently reviewed by two experts. Disagreements were resolved by consensus discussion between two of the investigators (SB and MV) in joint reading sessions. Ictal EEG and semiology features were extracted and documented using SCORE‐EEG.

In this retrospective study, EEG and semiology were analyzed at the level of clinically defined seizure types rather than fixed‐duration EEG segments. For each seizure, the entire semiology sequence and the first three observable ictal EEG patterns were reviewed and scored. Timing relative to seizure onset and exact seizure duration were not systematically recorded and were therefore not included in the analysis.

The following features were extracted and included in the analysis:

- Posterior dominant rhythm (PDR): symmetry/asymmetry and frequency.
- Interictal EEG: presence and localization of epileptiform discharges and of abnormal slowing.
- Ictal EEG: The onset pattern and the two earliest propagation patterns (when applicable). The ictal patterns were categorized as fast spike activity or repetitive spike activity, low‐voltage fast activity (LVFA), rhythmic activity (specifying frequency band and frequency in Hz), arrhythmic delta/theta activity, spike/sharp‐slow waves, or electrodecremental response (EDR). The electrodes (10–10 system nomenclature) where the activity was observed and the electrode(s) with the maximum voltage were reported.
- Ictal Semiology: Onset semiology feature (first that occurred), subsequent features in chronological order, and postictal signs, with lateralization where applicable. Semiology features and somatotopic descriptors were defined according to the ILAE Glossary.^20^ Ictal tachycardia was quantitatively defined as a doubling of the baseline heart rate.

All recorded seizures were reviewed per patient. Stereotypical seizures within a patient were scored together; different seizure types from the same patient were scored separately. All extracted features were exported to structured Excel spreadsheets for clustering analysis.

### 2.3 Data preprocessing and feature preparation

Prior to clustering, the EEG and semiology datasets were cleaned and standardized for analysis. Missing textual entries were replaced with empty strings to ensure consistent feature extraction. For semiology data, the original narrative descriptions were manually reviewed and cleaned up. The textual descriptions were then converted into numerical features using term frequency–inverse document frequency (TF–IDF) vectorization, producing continuous feature vectors that capture the relative importance of terms across the dataset.^21^ English stop words were removed during vectorization. The resulting TF–IDF matrices were high‐dimensional, with each feature corresponding to a term in the dataset. Because TF–IDF directly transforms text into continuous, normalized feature values, no additional scaling or encoding of categorical variables was required. These numerical representations were subsequently used as input for dimensionality reduction and clustering analyses. As multiple seizure types from the same patients were included, observations were not fully independent. The clustering approach did not explicitly account for this intrasubject correlation. However, single‐cluster sensitivity analyses were performed to assess the robustness of the clustering results.

### 2.4 Clustering analysis

Several unsupervised clustering approaches were explored, including both partition‐based and hierarchical methods. The unsupervised strategy was specifically chosen to allow the algorithm to discover latent seizure groupings without imposing predefined labels, with the aim of identifying clusters that are clinically meaningful. Models were compared using internal cluster validity metrics, including the silhouette score^22^ the Calinski–Harabasz index, within‐cluster and between‐cluster sum of squares (WSS and BSS), and their variance ratios (BSS/WSS and BSS/TSS).^23^ The optimal number of clusters for partition‐based methods was determined using the elbow method, complemented by silhouette analysis and visual inspection of the resulting cluster structure. Clustering was performed in the reduced‐dimensional space obtained after principal component analysis (PCA), with the first two principal components retained, and all internal validity metrics were computed in this same space. The silhouette score was calculated using Euclidean distance. Two types of visualizations were used to support interpretation of the clustering results: PCA scatterplots for partition‐based clustering (k‐means) and dendrograms for agglomerative hierarchical clustering. Visual inspection of the PCA plot refers to the qualitative assessment of the spatial distribution of individual seizures (represented as points) in the reduced‐dimensional space defined by the first principal components. Specifically, we examined whether seizures formed visually discernible groupings or gradients along the principal component axes. To aid interpretation, individual data points were mapped back to the original dataset and their associated seizure origin was evaluated. This post hoc annotation suggested a tendency for seizures of left temporal, right temporal, and extratemporal origin to occupy partially distinct regions of the PCA space. For hierarchical clustering, visual inspection of the dendrogram was used to evaluate the structure and separation of clusters. In this context, the vertical axis (“Distance”) represents the dissimilarity between clusters at the point of merging, with higher values indicating more distinct groups. The dendrogram was truncated by collapsing lower‐level branches representing individual observations to improve readability while preserving the higher‐level cluster structure. Particular attention was given to identifying large linkage distances between cluster merges, as these indicate meaningful separation between groups. Visual inspection of the dendrogram revealed clear separation between the main clusters, supporting the selection of three clusters, consistent with internal validation metrics such as the elbow method. The clinical relevance was then assessed as the accuracy for localizing at lobar level and lateralizing the seizures. The clustering methods that yielded the most clinically interpretable and coherent patterns were selected for further analysis.

#### 2.4.1 Overview of applied clustering methods

*K‐Means Clustering* is a widely used partitioning method that divides observations into a predefined number of groups (k) by minimizing variation within each cluster. The algorithm iteratively assigns each data point to the nearest cluster center and then updates these centers as the mean of the points in each group.^24^

*Agglomerative Hierarchical Clustering* is a bottom‐up hierarchical method that begins with each data point as its own cluster and iteratively merges the closest pairs based on a linkage criterion (e.g., Ward's, single, or average linkage). The process continues until a single cluster remains or a desired number of clusters is obtained.^24^

#### 2.4.2 EEG feature clustering

EEG narrative descriptions were converted into numerical features using TF–IDF vectorization after removal of English stop words and handling of missing values. This transformation produced a seizure‐by‐term feature matrix in which each narrative was represented as a vector of weighted term importance scores. Prior to adopting TF–IDF, we also evaluated a one‐hot encoding approach, where each token was treated as a binary feature indicating presence or absence. However, this representation produced unstable clusters and lacked clinical interpretability, whereas TF–IDF yielded more coherent and meaningful groupings.

Dimensionality reduction was performed using PCA^24^ to project the high‐dimensional TF–IDF feature matrix into a lower‐dimensional space prior to clustering. The first two principal components were retained to enable visualization of the data and cluster structure in a two‐dimensional PCA scatterplot. The number of components was not determined through formal optimization but was fixed to two to enable visualization and consistency with the plotted representation. These components together explained approximately 34% of the total variance. Inspection of the cumulative explained variance indicated that a substantially larger number of components (16 principal components) would be required to capture 80% of the variance, reflecting the high‐dimensional nature of the data. Therefore, the two‐component representation was primarily used for visualization and qualitative assessment, while clustering and internal validity calculations were performed in this reduced‐dimensional space.

**FIGURE 1:**
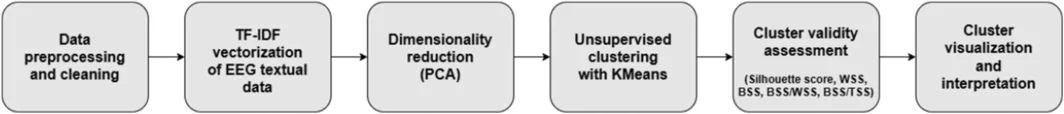
Overview of the EEG clustering workflow. EEG textual descriptions were first preprocessed and cleaned, then converted into numerical features using TF–IDF vectorization. The high‐dimensional feature matrix was reduced to two principal components using PCA, after which k‐means clustering was applied to identify groups of EEG patterns. Cluster quality was evaluated using internal validity metrics (silhouette score, WSS, BSS, BSS/WSS, BSS/TSS), and the resulting clusters were visualized and interpreted.

K‐means clustering was applied to the PCA‐transformed feature space, and the optimal number of clusters was determined using the elbow method.^22^ Cluster quality was evaluated using several internal validity metrics, including the silhouette score, WSS, BSS, and the ratios BSS/WSS and BSS/TSS. These complementary measures assess cluster compactness, separation, and the proportion of variance explained by the clustering structure. The EEG clustering workflow is illustrated in Figure 1.

#### 2.4.3 Semiology feature clustering

Semiology variables were preprocessed by combining the individual semiology fields into a single textual description for each seizure episode. These composite descriptions were converted into numerical feature vectors using TF–IDF representation. Because TF–IDF does not explicitly preserve temporal ordering, we additionally explored a sequential categorical representation using one‐hot encoding of ordered semiology stages. However, this alternative approach did not improve clustering performance and often produced clinically implausible solutions, including highly unbalanced clusters or clusters containing only one or two seizures. TF–IDF was therefore retained as the primary representation.

To reduce dimensionality and preserve the local structure of the semiology feature space, the TF–IDF matrix was projected into a lower‐dimensional embedding using Uniform Manifold Approximation and Projection (UMAP).^25^ Unsupervised clustering was performed in the UMAP embedding using three algorithms: k‐means, Density‐Based Spatial Clustering of Application of Noise (DBSCAN),^24^ and agglomerative hierarchical clustering. The clustering solutions were compared using the silhouette score. Agglomerative hierarchical clustering achieved the highest silhouette score and was therefore selected for further analysis. For comparison, the k‐means results are also reported in the manuscript, although they produced lower silhouette scores than the hierarchical approach. Inspection of the cumulative explained variance indicated that a large number of components (37 principal components) would be required to capture 80% of the variance, reflecting the high‐dimensional nature of the feature space. Therefore, the two‐component PCA representation was used primarily for visualization and qualitative assessment rather than comprehensive variance representation.

**FIGURE 2:**
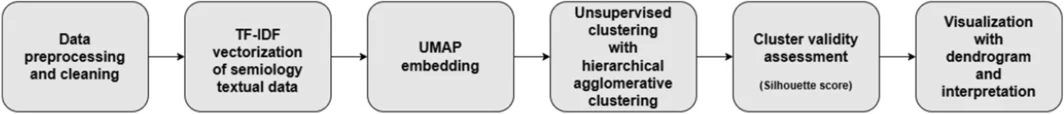
Overview of the semiology clustering workflow. Semiology data were preprocessed and textual descriptions were converted into numerical features using TF–IDF vectorization. The high‐dimensional feature space was reduced using UMAP, followed by hierarchical agglomerative clustering. Cluster quality was assessed with the silhouette score, and hierarchical relationships were visualized using a dendrogram.

Agglomerative hierarchical clustering was subsequently used to characterize the hierarchical relationships between seizures and to generate the dendrogram representation. For this purpose, distances between merged clusters were retained during model fitting and converted into a linkage matrix for dendrogram visualization. The semiology clustering workflow is presented in Figure 2.

#### 2.4.4 Combines EEG and semiology clustering

Additionally, we explored an integrated clustering framework combining EEG and semiology features to achieve a less fragmented analytical approach.

#### 2.4.5 Combining EEG, semiology, and integrated demographic data in clustering

In the primary analyses, EEG and semiology features were initially explored separately to allow the clustering algorithms to identify patterns without introducing predefined segmentation based on demographic characteristics. Demographic variables were therefore not included in the initial models in order to avoid biasing the clustering structure. Subsequently, we evaluated the potential contribution of demographic variables by repeating the clustering analyses after incorporating age and sex into all scenarios, including EEG only, semiology only, and combined EEG and semiology datasets.

### 2.5 Resected focus and outcome

Clusters were evaluated based on three clinical parameters obtained from surgical reports, postoperative MRIs, and follow‐up data: (1) Region of resection (temporal or extratemporal); (2) Side of resection (left or right); (3) Surgical outcome at 12 months, classified by the Engel Class Score (ECS): Class I = Free of disabling seizures; Class II = Rare disabling seizures; Class III = Worthwhile improvement; Class IV = No worthwhile improvement.^26^ For statistical analysis, outcomes were dichotomized into seizure‐free (ECS I) versus non–seizure‐free (ECS II–IV). As the main focus of this paper is on clinical benefit for the patients, we opted to dichotomize according to presence or absence of disabling seizures.

### 2.6 Conventional interpretation of electroclinical features

Independently from the clustering, and to allow comparison with the previously published papers, we evaluated the sensitivity, specificity, and overall accuracy of the ictal electroclinical features. For EEG, we extracted the region where the ictal EEG onset pattern was identified on the scalp. For semiology, we considered the following features as associated with temporal foci: epigastric aura, oroalimentary automatisms, gestural automatisms, any automatism (oroalimentary, gestural, vocal, or verbal), epigastric aura followed by automatisms, dystonic posturing, co‐occurrence of gestural automatisms with dystonic posturing.^20^ In addition, we considered the following lateralizing signs: gestural automatisms (ipsilateral), dystonic posturing (contralateral), aphasia (speech‐dominant hemisphere), early head turning (ipsilateral), version prior to secondary generalization (contralateral), partial responsiveness co‐occurring with automatisms (speech nondominant hemisphere), last clonic jerks (ipsilateral), and postictal nose‐wiping (ipsilateral).

### 2.7 Statistical analysis

Statistical analyses were performed using Stata/MP 18.5. Normality of continuous variables was assessed with Quantile–Quantile (QQ) plots; normally distributed data are reported as mean ± standard deviation (SD), and categorical variables as proportions.

Associations between clusters and the resected focus and outcome were evaluated using Pearson's chi‐squared test. When overall significance was detected, post hoc pairwise comparisons were conducted with chi‐squared or Fisher's exact tests, as appropriate. Bonferroni correction was applied for multiple comparisons in this part of the analysis (significance threshold = .05/*n* comparisons). Because some patients with multiple seizure types were assigned to more than one cluster, these analyses were performed twice^1^: including all patients, and^2^ excluding patients assigned to multiple clusters to control for potential bias.

To further investigate the potential impact of clusters on surgical outcomes, patients were stratified into three groups: (a) left temporal resection, (b) right temporal resection, and (c) extratemporal resection. Within each group, the frequency of seizure‐free patients (ECS I) in each cluster was compared to those outside that cluster, using Fisher's exact test. Proportions and corresponding 95% confidence intervals (CIs) were calculated. Logistic regression was performed on the entire dataset, independent of clustering, to explore whether individual electroclinical features were independently associated with surgical outcome (Engel Class I vs. non‐Class I). We used the McNemar test to compare the sensitivity and specificity (Material S1) of clustering and conventional interpretation, to localize temporal foci (at the level of side and lobe).

## 3 RESULTS

A total of 116 patients (56% female; mean age at surgery: 34.8 ± 15.2 years) were included, from whom 220 seizures (stereotypical seizure types) were analyzed. The median number of seizure types analyzed per patient was 2 (IQR 1–2, mean: 1.89). Most patients (91; 78.4%) underwent temporal lobe resection, while 24 (20.7%) had extratemporal surgery, and 1 (.9%) had combined resection. Among extratemporal surgeries, 9 (7.8%) were frontal, 2 (1.7%) parietal, 5 (4.3%) occipital, 5 (4.3%) insular, and 3 (2.6%) multilobar. Resective surgery was performed on the right in 46 cases (39.6%), on the left in 69 (59.5%), and bilaterally in one patient (.9%).

At 12 months, 83 patients (71.6%, 95% CI: 62.6–79.1) were seizure‐free (Engel Class I). Of the remaining 33 patients (28.4%, 95% CI: 20.9–37.4), 17 (14.6%) had Engel II, seven (6.0%) Engel III, and nine (7.8%) Engel IV outcomes. Among patients who underwent temporal lobe resection 76.9% (95% CI: 60.0–98.2%) were seizure‐free (Engel Class I).

### 3.1 Clustering analysis

The integrated approaches fusing EEG and semiology, with and without demographic data, did not yield better or clinically meaningful clustering (Material S2). Therefore, analyses presented in the main text focus on the separate EEG and semiology clustering results derived from the original feature sets.

#### 3.1.1 EEG clusters

K‐means clustering identified three EEG clusters (EEG‐1, EEG‐2, EEG‐3), based on the elbow method. The clustering solution achieved a silhouette score of .63. While this value falls within the range traditionally described as indicating a reasonable clustering structure according to the criteria of Kaufman and Rousseeuw,^22^ in the context of heterogeneous clinical datasets, it suggests good and comparatively strong cluster separation.^27^ Additional cluster validity metrics further supported the robustness of the clustering solution. The WSS (7.97) indicated relatively compact clusters, while the BSS (44.84) suggested substantial separation between clusters. The ratio of between‐cluster to within‐cluster variance (BSS/WSS = 5.63) further indicated strong separation relative to internal cluster variability. Moreover, the proportion of total variance explained by the clustering (BSS/TSS = .85) showed that approximately 85% of the variance in the data was attributable to differences between clusters rather than variability within clusters, supporting the stability of the identified cluster structure. The Calinski–Harabasz index (597) further indicated strong cluster separation and compactness.

**FIGURE 3:**
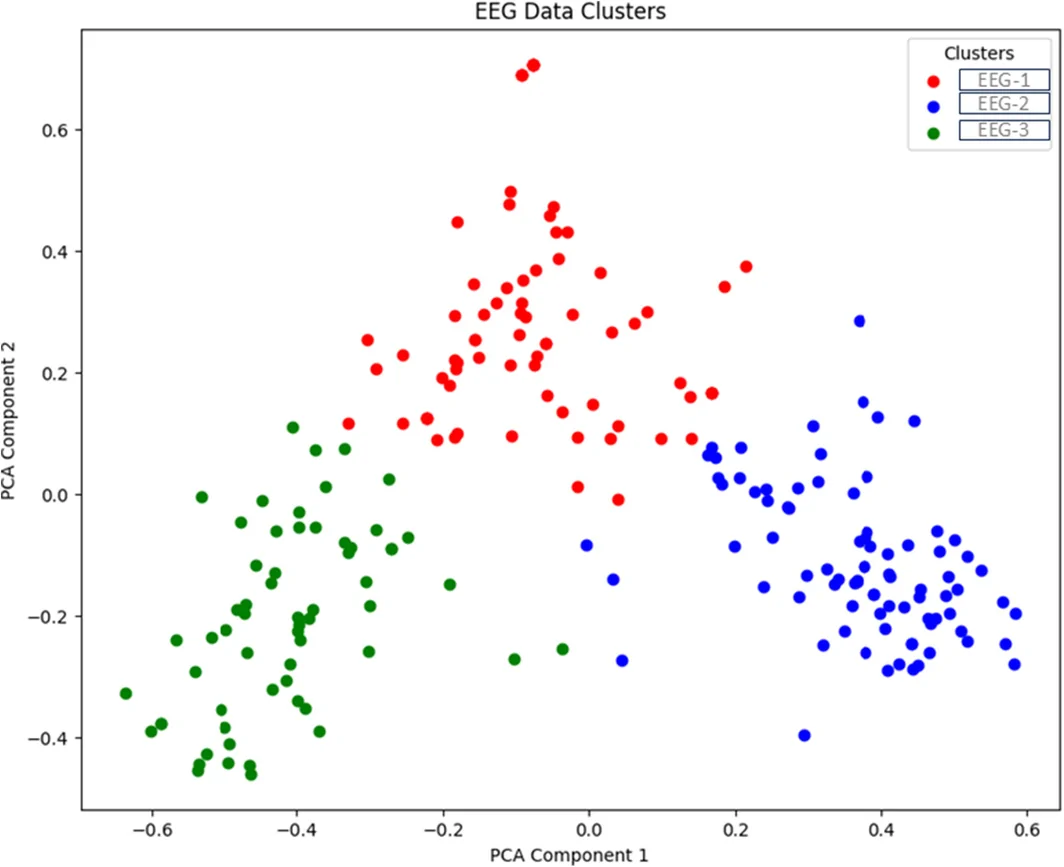
Principal Component Analysis (PCA) plot of EEG clusters identified by k‐means clustering. K‐means clustering was applied with the number of clusters set to three (k = 3), as determined by the elbow method, resulting in three EEG clusters (EEG‐1, EEG‐2, EEG‐3). Each point represents a seizure and is colored according to its assigned cluster. The clustering solution achieved a silhouette score of .63, indicating good separation between clusters according to commonly used criteria. Additional internal validity metrics supported the robustness of the clustering structure, including relatively low within‐cluster dispersion (WSS = 7.97), substantial between‐cluster variance (BSS = 44.84), and a high proportion of variance explained by cluster separation (BSS/TSS = .85). The Calinski–Harabasz index (597) further indicated strong cluster separation and compactness. In total, 141 cluster assignments were derived from 91 unique patients. The plot shows the distribution of clusters in the two‐dimensional PCA space used for visualization; however, dimensionality reduction may obscure relationships present in the original high‐dimensional feature space.

Twenty‐five patients had seizures assigned to two clusters, while no patient was assigned to all three clusters. The EEG‐1 cluster included 36 seizures (16 unique patients), EEG‐2 included 59 (41 unique patients), and EEG‐3 included 46 (34 unique patients). In total, 141 cluster assignments were derived from 91 unique patients. A PCA visualization of the clusters is presented in Figure 3. It is important to note that PCA reduces high‐dimensional data to two components, which may obscure or distort relationships present in the original feature space.

The resection sites and outcome in the three EEG clusters are summarized in Table 1.

**TABLE 1:** Distribution of surgery side, region, and outcome in each of the three EEG clusters.

		EEG clusters		
		EEG‐1	EEG‐2	EEG‐3
Side of resection	Left	20 (55.6%)	58 (98.3%)	9 19.6%
	Right	15 (41.7%)	1 (1.7%)	37 (80.4%)
	Bilateral	1 (2.8)	0 (0)	0 (0)
Region of resection	Temporal	18 (50%)	55 (93.2%)	40 (86.9%)
	Extratemporal	18 (50%)	4 (6.8%)	5 10.9%
	Temporal+	0 (0)	0 (0)	1 (2.2)
Outcome	ECS I	26 (72.2%)	44 (74.6%)	29 (63.0%)
	ECS not I	10 (27.8%)	15 (25.4%)	17 (37.0%)
	Total	36 (100%)	59 (100%)	46 (100%)

The distribution of resection side differed significantly across EEG clusters (overall *χ* ^2^, *p* < .001). Cluster EEG‐2 was predominantly associated with left‐sided resections, while Cluster EEG‐3 included mostly right‐sided surgeries (80.4%, *p* < .001). Cluster EEG‐1 exhibited a near‐equal distribution of right‐ (41.7%) and left‐sided (55.6%) resections and differed significantly from both Cluster EEG‐2 and EEG‐3 (*p* < .001 for both comparisons). These significant differences between the clusters were also found among patients assigned to a single cluster (Material S3).

Regarding the surgical region, Clusters EEG‐2 and EEG‐3 consisted mainly of patients with temporal lobe resections (93.2% and 87.0%, respectively), whereas Cluster EEG‐1 included a higher proportion of extratemporal surgeries (50%). The overall difference in resection region across clusters was significant (overall *χ* ^2^, *p* < .001), with EEG‐1 differing significantly from both EEG‐2 and EEG‐3 (*p* < .001), while no significant difference was found between EEG‐2 and EEG‐3 (*p* = .387). In the subgroup of patients assigned to a single cluster (Material S3), results were similar for EEG‐2 and EEG‐3, and more pronounced for EEG‐1, that comprised 93.8% extratemporal cases and differed significantly from the other two clusters (*p* < .001).

The proportion of patients achieving Engel Class I at 12 months did not differ significantly across clusters (overall *χ* ^2^, *p* = .42). Cluster EEG‐3 had a slightly lower rate of favorable outcomes (63%) compared to EEG‐2 (72.2%) and EEG‐1 (74.6%), as well as the overall cohort (71.6%), but these differences were not statistically significant (*p* = .203, .38, and .31, respectively). Results were similar when restricting the analysis to patients in a single cluster (Material S3).

The analysis for the resection site was repeated in the subgroup of patients with ECS I outcomes. Again, significant differences were observed across clusters for both resection side and region (*p* < .001 in all comparisons). All pairwise comparisons for resection side were statistically significant, while for the resection region, EEG‐1 differed significantly from EEG‐2 and EEG‐3. Detailed results are provided in Material S3.

To further characterize cluster‐specific features, we identified seizures corresponding to the central data points of each cluster and those at the transition zones between clusters, based on the PCA plot.

##### EEG cluster characteristics

*Cluster EEG‐1* was characterized by central datapoints corresponding to extratemporal ictal onsets, predominantly in frontal and central regions, with the parieto‐occipital regions being the second most common, with no clear lateralization. Peripheral datapoints transitioning toward Clusters EEG‐2 and EEG‐3 showed additional early involvement of left or right temporal electrodes, respectively. Temporal involvement appeared either at onset, typically in frontotemporal or post‐temporoparietal regions, or emerged following an initial EEG period with no observable change. A wide range of initial ictal EEG patterns were seen, including spike/sharp‐slow wave activity, fast or repetitive spikes, LVFA, arrhythmic delta/theta activity often mixed with spikes and rhythmic activity. The most frequent, observable initial ictal pattern across the full cohort and the patients assigned to single clusters was rhythmic delta activity and LVFA.

*Cluster EEG‐2* was defined by central datapoints showing maximal ictal voltage at the left temporal electrodes. Peripheral datapoints adjacent to Cluster EEG‐3 reflected early right temporal spread or independent right temporal ictal activity, while those near Cluster EEG‐1 indicated early extratemporal propagation alongside dominant left temporal involvement. Rhythmic delta and theta activity was the predominant initial ictal pattern, followed by irregular delta/theta activity and LVFA. These findings were consistent across the entire cluster and after excluding patients present in multiple clusters.

*Cluster EEG‐3* had central datapoints corresponding to seizures with maximal ictal activity in the right temporal electrodes. Peripheral datapoints adjacent to Cluster EEG‐1 showed additional extratemporal involvement, while those near Cluster EEG‐2 reflected early left temporal spread. Rhythmic activity, primarily in theta or alpha frequency bands, was the most common initial ictal pattern, followed by arrhythmic delta/theta activity. This pattern was consistent across the entire cluster and the subset of unique patients.

#### 3.1.2 Semiology clusters

**FIGURE 4:**
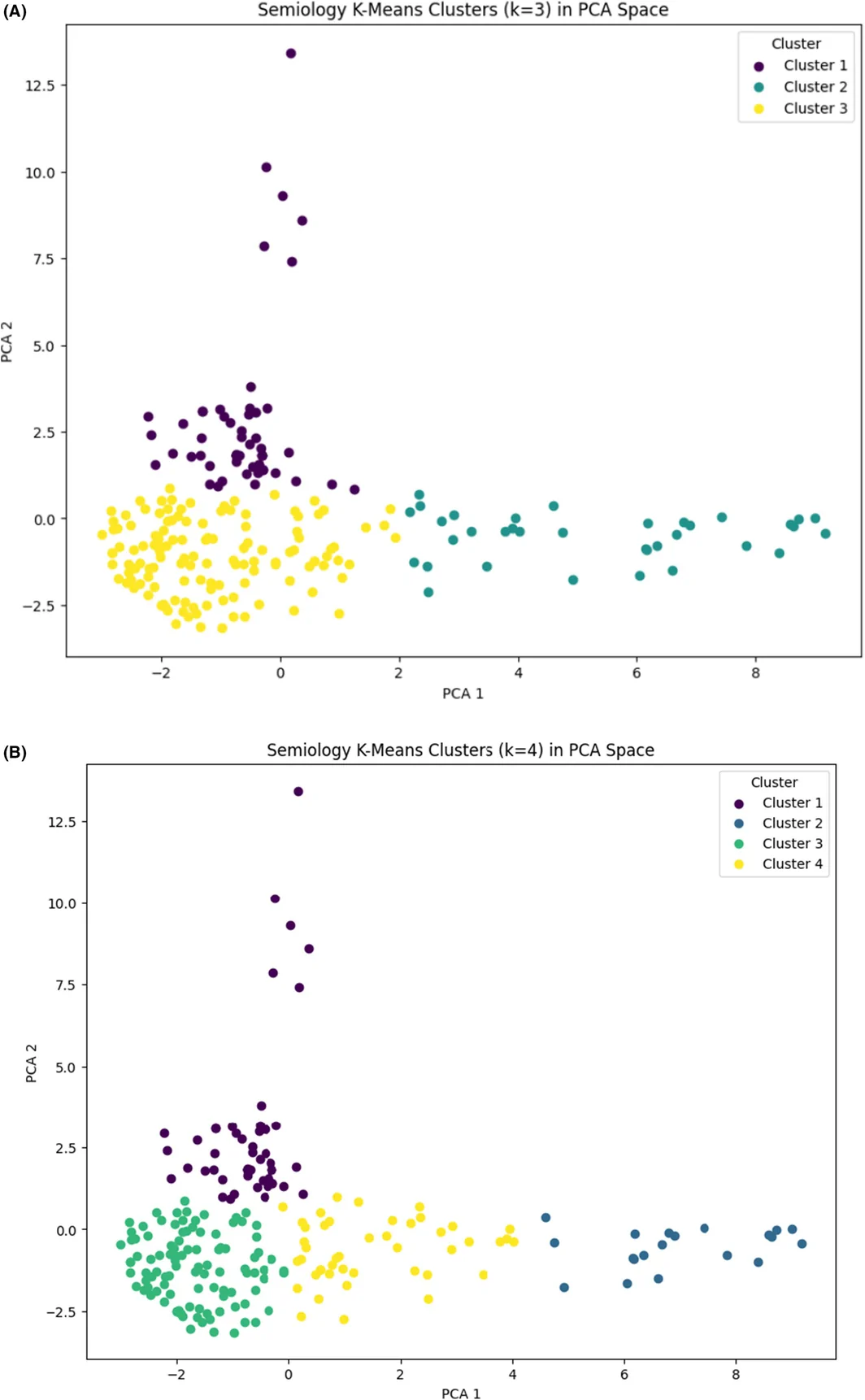
(A) Principal Component Analysis (PCA) visualization of semiology clusters obtained using k‐means clustering with three groups (k = 3). The clusters show relatively clear separation along the first principal component, particularly for the group located on the right side of the plot. Some overlap remains between the two clusters on the left, but the overall structure suggests reasonably coherent grouping. The clustering solution achieved a silhouette score of .48, indicating moderate cluster separation. (B) Principal Component Analysis (PCA) visualization of semiology clusters derived from k‐means clustering with four groups (k = 4). Some separation between clusters is observable; however, several points overlap across cluster boundaries. This pattern suggests only moderate cluster distinctiveness, consistent with the relatively modest silhouette score (.47).

**FIGURE 5:**
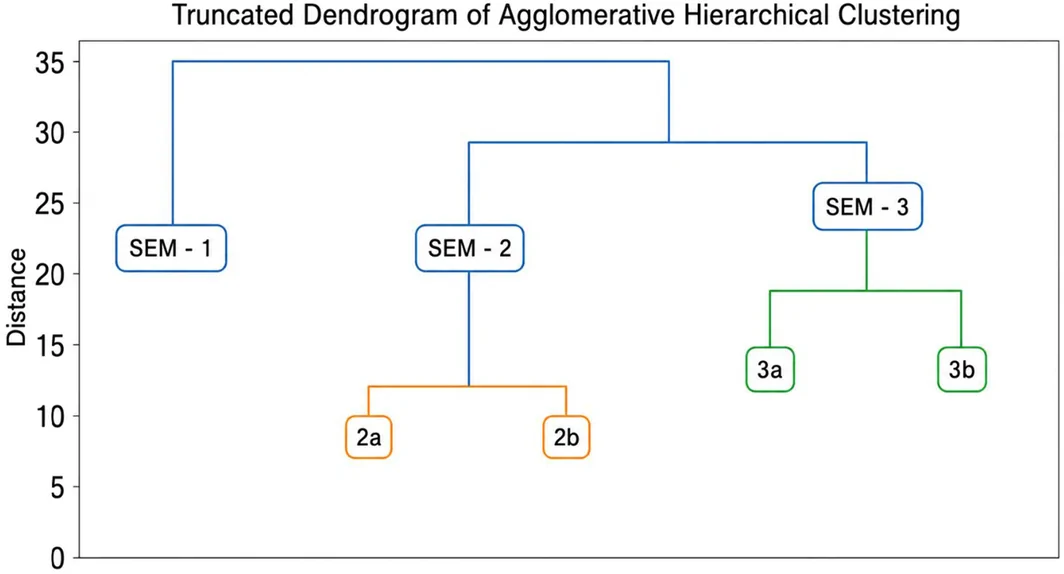
Truncated dendrogram obtained from agglomerative hierarchical clustering of semiology data. Lower‐level branches representing individual observations were collapsed to improve readability while preserving the higher‐level cluster structure. The dendrogram shows three main clusters (SEM‐1, SEM‐2, SEM‐3) and five subclusters (SEM‐1, SEM‐2a, SEM‐2b, SEM‐3a, SEM‐3b). The *y*‐axis (“Distance”) represents the dissimilarity between clusters at the point of merging, with higher values indicating more distinct groups.

The clustering analysis using the k‐means algorithm was first explored with both three and four clusters (k = 3 and k = 4). These solutions yielded similar silhouette scores of .48 and .47, respectively, indicating only weak to moderate cohesion and separation between clusters.^20^ While this method provided an initial grouping structure (Figures 4 and 5), the relatively modest silhouette scores and the complex, polymorphic, and hierarchically structured nature of ictal semiology suggested that alternative unsupervised clustering approaches should be explored.

We therefore evaluated additional unsupervised clustering methods, including DBSCAN and agglomerative hierarchical clustering. Among these approaches, agglomerative hierarchical clustering achieved the highest silhouette score (.54), indicating improved cluster cohesion and separation compared to the k‐means solutions. The hierarchical algorithm initially identified 23 fine‐grained clusters, which were subsequently merged into five subclusters and ultimately into three primary clusters (Figure 5). Visual inspection of the dendrogram revealed large linkage distances separating the three main clusters, supporting their selection as a well‐separated and interpretable grouping of seizure semiology patterns, consistent with the elbow method and other internal validation metrics.

To improve readability of the hierarchical structure, the dendrogram was truncated by collapsing lower‐level branches representing individual observations. This allowed clearer visualization of the higher‐level cluster relationships while preserving the main hierarchical structure and emphasizing the three principal clusters identified in the analysis.

The five subclusters included 169 observations (72 unique patients), while the three main clusters comprised 150 observations (85 unique patients). Proceeding with the three‐cluster solution, SEM‐1 included 16 seizures (four unique patients), SEM‐2 included 73 (47 unique patients), and SEM‐3 included 61 (34 unique patients). Thirty‐one patients were represented in multiple clusters, including three patients assigned to all three clusters.

There was a significant overall difference in the region of surgery across the semiology clusters (*χ* ^2^, *p* = .002). SEM‐2 consisted almost exclusively of temporal lobe resections (91.8%), compared to 68.8% and 65.6% in SEM‐1 and SEM‐3, respectively. Most extratemporal resections were found in SEM‐3. Differences in resection side and surgical outcome were not statistically significant (*p* = .132 and *p* = .828, respectively). When restricting the analysis to patients assigned to a single cluster, the difference in resection region remained significant (*p* = .001), while side and outcome remained nonsignificant (*p* = .088 and *p* = .321). The distribution of region, side, and outcome per cluster is shown in Table 2.

**TABLE 2:** Proportions of resection side, region, and outcome within semiology clusters.

		Agglomerative semiology clusters		
		SEM‐1	SEM‐2	SEM‐3
Side of resection	Left	10 (62.5%)	50 (68.5%)	29 (47.5%)
	Right	6 (37.5%)	23 (31.5%)	31 (50.8%)
	Bilateral	0 (0)	0 (0)	1 (1.6%)
Region of resection	Temporal	11 (68.8%)	67 (91.8%)	40 (65.6%)
	Extratemporal	5 (31.3%)	5 (6.9%)	21 (34.4%)
	Temporal+	0 (0)	1 (1.8%)	0 (0)
Outcome	ECS I	10 (62.5%)	50 (68.5%)	43 (70.5%)
	ECS not I	6 (37.5%)	23 (31.5%)	18 (29.5%)
	Total	16 (100%)	73 (100%)	61 (100%)

Pairwise comparisons for resection region, adjusted with Bonferroni correction, revealed a significant difference only between SEM‐2 and SEM‐3 (*p* < .001), both in the full cohort and among patients assigned to single‐cluster.

The same analysis of resection side and region was repeated for patients with ECS I outcome. Unlike the previous analysis, including all patients, a significant overall difference in surgery side distribution emerged (*p* = .04 for all patients; *p* = .035 for single‐cluster patients). Pairwise comparison between SEM‐2 and SEM‐3 yielded a *p*‐value of .023 (all patients), thus, not significant after Bonferroni correction. However, the difference was significant when restricted to single‐cluster patients (*p* = .012). As in the full cohort, a significant overall difference in resection region distribution was observed (*p* = .002 and *p* = .001 for all and single‐cluster patients, respectively), with SEM‐2 and SEM‐3 differing significantly (*p* < .001 in both analyses). Further details are provided in Material S4.

##### Semiology cluster characteristics

The five most frequent semiology features in each cluster are shown in Table 3, based on both the full cohort and the subgroup of patients assigned to single‐cluster.

**TABLE 3:** Five most common semiology features per cluster, when including the entire cluster and only patients assigned to single clusters.

SEM‐1 cluster		SEM‐2 cluster		SEM‐3 cluster	
Entire, 18 seizures	Unique pt., five seizures	Entire, 111 seizures	Unique pt., 74 seizures	Entire, 91 seizures	Unique pt., 56 seizures
Bilat tonic–clonic with figure‐of‐4 (18; 100%)	Bilat tonic–clonic with figure‐of‐4 (5, 100%)	Oroalimentary automatisms (81, 73%)	Oroalimentary automatisms (56; 75.7%)	Tonic (36; 39.6%)	Ictal tachycardia (22; 39.3%)
Head version (14; 77.8%)	Head version (4; 80%)	Manual automatisms distal (60; 54.1%)	Manual automatisms distal (39; 52.7%)	Head version (36; 39.5%)	Tonic (21; 37.5%)
Tonic (13; 72.2%)	Tonic (4; 80%)	Ictal tachycardia (46; 41.4%)	Ictal tachycardia (32; 48.6%)	Ictal tachycardia (29; 31.9%)	Head version (19; 33.9%)
Oroalimentary automatisms (11; 61.1%)	Oroalimentary automatisms (4; 80%)	Postictal aphasia (34; 30.6%)	Postictal aphasia (24; 32.4%)	Bilateral tonic–clonic (29; 31.9%)	Hypermotor behavior (13; 23.2%)
Asymmetric last clonic jerks (8; 44.4%)	Asymmetric last clonic jerks (3; 60%)	Dystonic (34; 30.6%)	Postictal nose‐wiping (22, 29.7%)	Epigastric aura (23; 25.3%)	Bilateral tonic–clonic (11; 19.6%)

All 18 seizures in SEM‐1 were focal to bilateral tonic–clonic seizures (FBTCS), with 100% presenting the figure‐of‐four sign. Thirteen of the 16 patients had resections contralateral to the extended upper extremity, and 53.8% of these patients were seizure‐free, with a higher proportion of temporal lobe resections among seizure‐free patients (87.5%) compared to those not seizure‐free (50%). SEM‐2 comprised 111 seizures, with 70.3% focal impaired consciousness seizures (FIC). SEM‐3 included 91 seizures, predominantly focal preserved consciousness seizures (FPCS) (37.4%) and FBTCS (31.9%). When splitting cluster SEM‐3 up into the two identified subclusters 3a and 3b (composed of 48 and 43 seizures, respectively), the distribution of seizure types changed; subcluster 3a had 64.6% FPCS and only 6.3% FBTCS, while subcluster 3b had 60.5% FBTCS and 6.9% FPC. FBTCS in SEM‐3 showed the figure‐of‐four sign in only 10.3% of cases, unlike SEM‐1. Seizure type distribution differed significantly across clusters (*χ* ^2^_(6)_, *p* < .001; Cramér's V ≈ .65), indicating a large effect size. Similarly, the figure‐of‐four sign demonstrated a highly significant association with cluster membership (Fisher's exact test, *p* < .001; Cramér's V ≈ .60). Oroalimentary automatisms also differed significantly between clusters (*χ* ^2^_(2)_, *p* < .001; Cramér's V ≈ .38). Further details on the semiology clusters and on the potential influence of the clusters (EEG and semiology) on the outcome are provided in Material S5 and S6, respectively.

#### 3.1.3 Accuracy of the clusters, compared with conventional interpretation

Because the majority of the operated patients in our cohort were temporal resections, we determined the accuracy of the clustering and of the conventional interpretation to identify temporal EZ (including its lateralization). As described in detail earlier, EEG cluster 1 corresponded to extratemporal EZ, EEG cluster 2 indicated left temporal, and EEG cluster 3 indicated right temporal EZs. The accuracy of the clustering and of the conventional assessment is detailed in Table 4. There was no statistically significant difference in diagnostic performance between the EEG clustering and the conventional interpretation of ictal EEG onset.

**TABLE 4:** Accuracy of the EEG clusters as compared with the conventional interpretation.

	EEG clustering	Ictal onset
Sensitivity (95% CI)	73.6% (61.9–83.3)	80.0% (68.7–88.6)
Specificity (95% CI)	42.3 (23.6–63.1)	28.6% (11.3–52.2)
Overall accuracy (95% CI)	65.3 (55.0–74.6)	68.1% (57.5–77.5)

The semiology clusters were not suitable for localizing the EZ: there were no significant differences in lateralization, and although lobar localization was statistically significant at group level, the considerable overlap precluded a meaningful clinical application. The conventional interpretation of temporal lobe semiology along with the lateralization signs gave a sensitivity of 87.3% (95%CI: 77.3%–94.0%), specificity of 21.7% (95% CIs: 7.5%–43.7%) and overall accuracy of 71.3% (61.0%–80.1%). Further details on diagnostic accuracy of the individual semiology features, as well as the lateralization value of semiology features are presented in Material S7A,B, respectively.

## 4 DISCUSSION

Unsupervised clustering of EEG and semiology features did not improve localization and lateralization of the EZ, compared with conventional clinical interpretation, in temporal lobe epilepsy.

Was our cohort representative? Out of the 116 patients in our cohort, 71.6% achieved an Engel Class I outcome. This aligns with previously published international results.^1^, ^2^, ^28^ Patients who underwent extratemporal resections had lower odds of achieving Engel Class I at 12 months postoperatively, a finding consistent with prior literature.^1^ The majority of patients (78.4%) underwent temporal lobe resections, reflecting the predominance of temporal lobe epilepsy among surgical candidates.^29^, ^30^ Due to the relatively limited number of extratemporal cases, we focused our analysis on localizing the EZ to the temporal lobe and lateralizing it.

Clustering based on ictal EEG features identified three clusters, with a strong silhouette score of .63,^22^, ^27^ suggesting reasonably distinct yet not totally separated groupings.^22^ Indeed, while some overlap occurred (some patients presented seizures with different patterns, different onset sites, and propagation pathways resulting in the same patient being assigned to several clusters), we found significant differences in relation to the side and region of surgery, between the clusters. This demonstrates that the EEG clusters are clinically meaningful. When restricting the analysis to patients assigned to a single cluster, EEG‐1 was composed almost exclusively of extratemporal cases; cluster EEG‐2 was predominantly associated with left temporal resections, while the EEG‐3 cluster included primarily right temporal resections.

The greatest heterogeneity in initial ictal EEG patterns was observed in cluster EEG‐1, which included a range of electrographic features such as sharp or spike–wave discharges, fast spike or repetitive spike activity, LVFA, and arrhythmic or rhythmic delta/theta activity. This diversity likely reflects the electrographic complexity and widespread networks, characteristic of extratemporal epilepsies.^31^ The frequent occurrence of nonobservable ictal EEG onset in this cluster suggests that the epileptogenic focus may lie in regions not optimally sampled by scalp electrodes, or that the initial EEG changes are of low amplitude or masked by background activity.

Although extratemporal resections were generally associated with lower odds of seizure freedom in the overall cohort, patients within EEG‐1 cluster, comprising the highest proportion of extratemporal cases and, when restricted to single‐cluster assignments, almost exclusively extratemporal cases, had a relatively favorable surgical outcome. This finding implies that poor postoperative outcomes in extratemporal epilepsy may be driven by specific subgroups with distinct characteristics. One such characteristic appears to be the presence of a nonobservable ictal EEG onset: among the 13 patients in EEG‐1 cluster who exhibited seizures without an identifiable EEG onset, three underwent extratemporal resections, and none achieved seizure freedom. Additionally, the subgroup analysis revealed that extratemporal patients assigned to EEG cluster 2 had lower odds of achieving Engel Class I outcome. Retrospective review of their ictal EEG recordings demonstrated considerable variability in seizure onset patterns. Three of the four extratemporal cases in this cluster exhibited multiple seizures with divergent onset localizations, including combinations such as left temporal and parietal onsets following a period of nonobservable EEG change, or seizures involving both frontal and temporal regions. In one patient, a frontal onset was followed by rapid propagation to the left temporal lobe. These findings point to the presence of complex, and potentially multifocal or widespread, epileptogenic networks, which may underlie the poorer postoperative outcomes in this subgroup.^32^ Collectively, these results highlight the importance of evaluating ictal EEG heterogeneity and propagation patterns during presurgical assessment, particularly in patients considered for extratemporal resections.

Similarly, patients who underwent left temporal resections generally achieved favorable outcomes, with seizure freedom rates exceeding those of the overall cohort. However, subgroup analysis revealed that among these patients, those assigned to cluster EEG‐3 were less likely to achieve ECS I outcome compared to those outside this cluster. Retrospective review of their ictal EEG data indicated frequent patterns of bilateral temporal involvement or left‐sided seizure onset with rapid propagation to the right temporal lobe.

Rhythmic ictal activity was more frequently observed in EEG clusters EEG‐2 and EEG‐3, which predominantly included patients who underwent temporal lobe resections. This observation is consistent with previous findings indicating that temporal lobe seizures, particularly those of mesial origin, are commonly associated with rhythmic ictal discharges, likely reflecting the organized network dynamics of limbic structures.^33^

The semiology‐based clusters exhibited limited differentiation with respect to resection side and did not demonstrate significant associations with surgical outcome, either in the overall cohort or in subgroup analyses. The silhouette score for the agglomerative clustering based on semiology was moderate (.54).^22^ This lower clustering performance likely reflects the rapid propagation, as well as the relatively small sample size, complicating the identification of robust clusters predicting clear resection sites and outcomes, despite the well‐established clinical importance of semiology in the presurgical evaluation.^34^, ^35^

The high frequency of the figure‐of‐four sign in cluster SEM‐1—present in all 18 FBTCS within this cluster—contrasted with its low occurrence in clusters 2 and 3 (six cases in total), suggesting that this feature may have influenced the clustering. The figure‐of‐four sign is a well‐established lateralizing feature, typically indicating an epileptogenic focus contralateral to the extended arm.^36^, ^37^ In SEM‐1, 13 of 16 patients with this sign underwent resections on the contralateral hemisphere, supporting its expected lateralizing value.

Epigastric aura was, as expected, more commonly seen in temporal lobe seizures.^20^ Epigastric aura, especially when not followed by automatisms, has also been reported in extratemporal epilepsies, mainly in frontal lobe and insula seizures.^20^ In accordance with the literature, in our cohort, 25 of 27 patients with epigastric aura had temporal lobe epilepsy, while only two were extratemporal (one had resection at the insula and one at the frontal area). Facial, vocal, or oroalimentary automatisms were seen in seizures of the frontal lobe epilepsy patient. Both extratemporal cases with epigastric aura were classified outside the SEM‐2 cluster, which mainly included temporal lobe epilepsy patients with FIC seizures. When not followed by automatisms, epigastric aura was mainly seen as part of FPC seizures in cluster SEM‐3 (subcluster 3a).

The EEG clusters showed similar localization accuracy to the conventional assessment of ictal electroclinical features (EEG onset and semiology). Although the sensitivity of the conventional electroclinical interpretation was good (80%–87%), the specificity was rather low (21%–28%), and it was not improved by data‐driven clustering. This suggests that the limitation is intrinsic to the analyzed phenomena: the rapid propagation of ictal activity and the silent brain areas that do not generate semiology or scalp EEG signals elude the electroclinical analysis.

Several limitations should be acknowledged. Cluster‐based subgrouping inherently reduced the sample size, particularly when subgroup analyses were conducted according to surgical category. This resulted in wide CIs, reflecting statistical uncertainty and limiting the robustness of the findings. Thus, the results on the potential role of clusters on outcome should be cautiously interpreted. Patient overlap across clusters added further complexity. Additionally, a limitation of the TF–IDF representation is that it treats semiological features as independent and unordered, and therefore does not preserve the temporal sequence of manifestations, which may provide important information for the seizure onset zone. Repeated seizures with stereotyped semiology within a patient were represented once to avoid overweighting patients with more recorded seizures. When a patient showed clinically distinct seizure types, each seizure type was entered as a separate semiological observation because clustering was performed at the level of seizure semiology rather than at the patient level. We acknowledge that seizure types from the same patient are not fully independent; therefore, patient‐level clustering was considered in interpretation/assessed in sensitivity analyses.

A further limitation is the risk of type II error. Given the paired design (McNemar's test), the minimum detectable absolute difference was approximately 9%–22% for sensitivity and 30%–40% for specificity, depending on discordance rates. Smaller differences may therefore not have been detected, although such differences are unlikely to be clinically meaningful.^38^, ^39^

Nonetheless, the use of standardized data via the SCORE framework enhanced reproducibility and reduced inter‐rater variability. Although no strong associations were identified between clusters and outcomes, findings suggest that patients with heterogeneous EEG patterns or complex propagation, particularly in extratemporal epilepsy, may have poorer prognoses. This study demonstrates the feasibility of applying unsupervised learning to standardized electroclinical data and provides a foundation for future multicenter, large cohort studies to validate and expand upon these exploratory findings.

## AUTHOR CONTRIBUTIONS

Study conception and design, EEG and semiology review: Sándor Beniczky, Maria Vlachou. Data collection: Sándor Beniczky, Maria Vlachou, Helene Kaas, Guido Rubolli, Lars Pinborg. Data analysis: Bernadett Mólnar. Statistical Analysis: Maria Vlachou. Drafting the manuscript: Maria Vlachou, Bernadett Mólnar, Sándor Beniczky. Critical revision of the manuscript: all authors. Approval of final manuscript all authors.

## FUNDING INFORMATION

This research received no specific funding.

## CONFLICT OF INTEREST STATEMENT

The authors declare no conflict of interest related to this paper.

## PATIENT CONSENT

Patient consent was waived due to the retrospective nature of the study and the use of anonymized data.

**Test yourself**

1. What was the main conclusion of the study?
  - Unsupervised clustering clearly outperformed conventional electroclinical interpretation
  - Semiology clustering was superior to EEG clustering for lateralization
  - Unsupervised clustering identified meaningful patterns but did not improve localization/lateralization compared with conventional interpretation
  - Combined EEG‐semiology‐demographic clustering provided the best clinical model
2. How did EEG clustering perform compared with conventional ictal EEG onset interpretation?
  - It was significantly more accurate
  - It was significantly less accurate
  - It showed comparable performance, with no statistically significant difference
  - It was not evaluated against conventional interpretation
3. Why were semiology clusters considered clinically limited for localization?
  - They showed no association with seizure type
  - They were too small to analyze statistically
  - They showed substantial overlap, especially for localization and lateralization
  - They were based only on postictal signs

*Answers may be found in the* *Supporting Information*

## Supporting information


Data S1

Data S2

## Data Availability

The data that support the findings of this study are available upon reasonable request from the corresponding author.
