# Supplementary material for: Unsupervised clustering of electroclinical features in temporal lobe epilepsy: A data‐driven approach
Source: Epileptic Disord. 2026 May 19;28(4):1236–51. doi: 10.1002/epd2.70283 (PMC13499273; doi:10.1002/epd2.70283)
Supplement: Supplementary file 1 [file EPD2-28-1236-s001.pdf]

<u>Supplementary Material</u>

Supplementary Material 1

Definitions of positive & negative patients for accuracy analyses.

1. *Regarding EEG clusters, the following definitions of positive (true & false) and negative (true & false) were made:*

<u>Positive:</u> “Left temporal resection patients in cluster EEG-2 only” and “Right temporal resection patients in cluster EEG-3 only”

If seizure-free (Engel Class I): true positive

If not seizure-free (not Engel Class I): false positive

<u>Negative:</u> “Temporal resection patients in cluster EEG-1-only” or “temporal resection patients in multiple clusters” or “Right temporal or extratemporal resection patients in cluster EEG-2 only” or “Left temporal or extratemporal resection patients in cluster EEG-3 only”.

If seizure-free (Engel Class I): false negative

If not seizure-free (not Engel Class I): true negative

2. *Regarding the first visible ictal EEG sign, the following definitions of positive (true & false) and negative (true & false) were made:*

<u>Positive:</u> “Left temporal resection patients with left temporal EEG-onset” and “Right temporal resection patients with right temporal EEG-onset”.

If seizure-free (Engel Class I): true positive

If not seizure-free (not Engel Class I): false positive

<u>Negative:</u> “Temporal resection patients with extratemporal EEG-onset” or “temporal resection patients with multilobar or bilateral or diffuse EEG onset”, or “Left temporal resection patients with Right temporal EEG-onset”, or “Right temporal resection patients with Left temporal EEG-onset, or “extratemporal resection patients with temporal EEG-onset”.

If seizure free (Engel Class I): false negative

If not seizure-free (not Engel Class I) true negative

3A. Regarding overall temporal lobe semiology performance, the following definitions were made:

<u>Positive:</u> “Left temporal resection patients with any temporal semiology and any lateralization to the Left and no lateralization to the Right”, or “Right temporal resection

patients with any temporal semiology and any lateralization to the Right and no lateralization to the Left"

If seizure-free (Engel Class I): true positive

If not seizure-free (not Engel Class I): false positive

Negative: "temporal resection patients with no temporal semiology", or "Left temporal resection patients with any temporal semiology and any lateralization to the Right", or "Right temporal resection patients with any temporal semiology and any lateralization to the Left", or "extratemporal resection patients with any temporal semiology".

If seizure-free (Engel Class I): false negative

If not seizure-free (not Engel Class I): true negative

3B. Regarding accuracy of individual semiology features for identifying temporal lobe epilepsy, the following definitions were made:

Positive: "temporal resection patients with the feature X"

If seizure-free (Engel Class I): true positive

If not seizure-free (not Engel Class I): false positive

Negative: "temporal resection patients without the feature X" or extratemporal resection patients with the feature X"

If seizure-free (Engel Class I): false negative

If not seizure-free (not Engel Class I): true negative

Supplementary Material 2

Alternative Clustering Approaches.

Several alternative clustering strategies were explored to evaluate whether different feature representations, dataset combinations, and model specifications could improve cluster separation and interpretability. The corresponding validation metrics are presented in the accompanying supplementary tables.

Combined EEG and semiology clustering: An integrated clustering framework combining EEG and semiology features was evaluated to assess whether joint modeling could reveal additional structure in the data. However, clustering of the combined dataset consistently produced low silhouette scores and substantial cluster overlap. Additional validation metrics—including within-cluster sum of squares (WSS), between-cluster sum of squares (BSS), the ratio of BSS to WSS, and the proportion of total variance explained by BSS (BSS/TSS)—supported the silhouette analysis and did not indicate improved separation. Overall, the combined feature space appeared to introduce heterogeneity that obscured meaningful cluster structure.

Dimensionality reduction of seizure stages: To reduce model complexity, additional analyses were performed restricting the datasets to the first three EEG stages and the first three semiology stages. This dimensionality reduction did not improve clustering performance, as validation metrics remained low and cluster separation did not increase.

Inclusion of demographic variables: The analyses were repeated after incorporating age and sex. In the EEG dataset, clusters were largely defined by demographic variables rather than EEG characteristics, reducing the electrophysiological contribution to cluster differentiation. In the semiology dataset, inclusion of demographics resulted in weaker clustering performance compared with the original model, as reflected by lower validation metrics.

Alternative feature encoding strategy: Beyond the vectorization-based representation used in the primary analysis, a categorical encoding strategy using one-hot encoding was evaluated. For the EEG dataset without demographic variables, this approach yielded acceptable silhouette scores but produced clusters with substantial overlap and limited clinical interpretability.

In the semiology dataset, the categorical approach produced apparently favorable metrics but resulted in a clinically implausible clustering solution, with two clusters containing ≤2 seizures and most seizures grouped into a single cluster. Similar findings were observed for the combined EEG and semiology dataset, without meaningful improvement in cluster validity or interpretability.

Conclusion: Across all explored alternatives—including integrated EEG–semiology clustering, dimensionality reduction, inclusion of demographic variables, and categorical encoding—cluster separation and interpretability remained inferior to the primary analytical approach.

The different clustering approaches for EEG, semiology and combined dataset are presented in the tables below.

Table 2A. Clustering Approaches. EEG dataset.

Demographics	Encoding	k	Silhouette	WSS ↓	BSS ↑	BSS/WSS ↑	BSS/TSS ↑	Interpretation
Excl. age/sex	Vectorized	3	0.63	7.97	44.84	5.63	0.85	Best separation; slightly higher within-cluster variance than k=4
		4	0.59	5.68	47.13	8.30	0.89	Lower WSS and higher explained variance; possible over-partitioning
Excl. age/sex	One-hot	3	0.64	95.45	15.12	0.16	0.14	High silhouette but very low explained variance
		4	0.65	95.45	15.12	0.16	0.14	Minimal improvement; may isolate rare patterns
Incl. age/sex	Vectorized	3	0.37	519.0	581.0	1.12	0.53	Moderate clustering with considerable overlap
		4	0.36	437.1	662.9	1.52	0.60	Higher variance explained but no

								improvement in separation
Incl. age/sex	One-hot	3	0.43	226.0	322.3	1.43	0.59	Moderate separation with notable dispersion
		4	0.49	153.5	394.8	2.57	0.72	Best structure when demographics included

Table 2B. Clustering Approaches. Semiology Dataset

Demographics	Encoding	k	Silhouette (K-means)	Silhouette (Hier.)*	WSS ↓	BSS ↑	BSS/WSS ↑	BSS/TSS ↑	Interpretation
Excl. age/sex	Vectorized	3	0.48	0.538	889.5	1768.02	1.99	0.66	Moderate separation
		4	0.47		667.3	1990.3	2.98	0.75	Similar separation with higher explained variance
Excl. age/sex	One-hot	3	0.71		402.47	2585.43	6.42	0.86	Favorable metrics but clusters not clinically meaningful (most seizures in 1–2 clusters)
		4	0.71		363.07	2624.82	7.23	0.87	Similar pattern; clusters

									dominated by few groups
Incl. age/sex	Vectorized	3	0.47	0.51	894.43	1768.48	1.98	0.66	Weaker cluster structure with more overlap
		4	0.45		679.12	1983.78	2.92	0.75	Slightly improved variance but overlapping clusters remain
Incl. age/sex	One-hot	3	0.34		20030.96	1887.53	0.09	0.09	Weak cluster structure; very low separation
		4	0.35	0.31	18632.24	3286.25	0.18	0.15	Marginal improvement but separation remains low

**We did not report the dispersion metrics for hierarchical clustering because these measures (WSS, BSS, and related ratios) are designed for centroid-based methods such as K-means, which explicitly minimize within-cluster variance. Since hierarchical clustering does not optimize this objective, these metrics can be misleading and are therefore not directly comparable across the two methods.*

Table 2C. Clustering Approaches. Combined EEG & Semiology Data

Demographics	Encoding	k	Silhouette	WSS ↓	BSS ↑	BSS/WSS ↑	BSS/TSS ↑	Interpretation
Excl. age/sex	Vectorized	3	0.0383	4185.83	422.10	0.101	0.092	Very weak cluster structure; combining EEG and semiology likely introduced heterogeneity obscuring

								meaningful structure
		4	0.0423	4008.64	599.29	0.150	0.130	Slight improvement but clusters remain poorly separated
Excl. age/sex	One-hot	3	0.0987	935.33	239.47	0.256	0.204	Marginally higher separation but still weak cluster structure
		4	0.0672	894.29	280.51	0.314	0.239	Minor variance improvement without clear cluster separation
Incl. age/sex	Vectorized	3	0.0881	4352.40	681.62	0.157	0.135	Including demographics did not improve cluster structure
		4	0.0530	4313.14	720.88	0.167	0.143	Slight increase in explained variance but separation remains weak
Incl. age/sex	One-hot	3	0.0986	1192.53	300.53	0.252	0.201	Low separation despite moderate variance ratios
		4	0.0577	1202.46	290.60	0.242	0.195	No meaningful improvement

								in clustering structure

*** For the combined dataset, we also tested clustering methods other than K-means, but they performed poorly as well, suggesting that the combined dataset does not form clear clusters. Therefore, we present only the K-means results here to illustrate the overall weakness of the clustering structure in the combined dataset.*

Supplementary Material 3

Table 3A. Side of Resection by EEG Cluster. A) Unique cluster patients, B) All patients with EC I, C) Unique Cluster patients with EC I

EEG Cluster	Side of Resection	A. Unique Cluster Only N (%)	B. Full Cohort with EC I N (%)	C. Unique Cluster Only with EC I N (%)
**EEG-1**	Right	8 (50%)	8 (30.8%)	5 (41.7%)
	Left	7 (43.8%)	18 (69.2%)	7 (58.3%)
	Bilateral	1 (6.2%)	0 (0)	0 (0)
**EEG-2**	Right	0 (0)	0 (0)	0 (0)
	Left	41 (100%)	44 (100%)	31 (100%)
	Bilateral	0 (0)	0 (0)	0 (0)
**EEG-3**	Right	31 (91.2%)	26 (89.7%)	23 (95.8%)
	Left	3 (8.8%)	3 (10.3%)	1 (4.2%)
	Bilateral	0 (0)	0 (0)	0 (0)
**Overall Chi^2**		**$p < 0.001$**	**$p < 0.001$**	**$p < 0.001$**

Table 3B. Side of Resection by EEG Cluster, pairwise comparisons. (Bonferroni-corrected significance threshold: $p < 0.0167$)

**Pairwise Comparisons (Resection Side)**	p-value (Unique Cluster patients, all outcomes)	p-value (all patients with ECS 1)	p-value (Unique Cluster patients with EC1)
EEG-1 vs EEG-2	<0.001	< 0.001	< 0.001
EEG-1 vs EEG-3	0.005	<0.001	0.001
EEG-2 vs EEG-3	<0.001	<0.001	<0.001

Table 3C. Region of Resection by EEG Cluster, A) Unique cluster patients, B) All patients with EC I, C) Unique Cluster patients with EC I.

EEG Cluster	Region of Resection	A. Unique Cluster Only N (%)	B. Full Cohort with EC I N (%)	C. Unique Cluster Only with EC I N (%)
**EEG-1**	Temporal	1 (6.2%)	15 (57.7%)	1 (8.3%)
	Extratemporal	15 (93.8%)	10 (42.3%)	11 (91.7%)
	Combined	0 (0)	0 (0)	0 (0)
**EEG-2**	Temporal	39 (95.1%)	44 (100%)	31 (100%)
	Extratemporal	2 (4.9%)	0 (0)	0 (0)
	Combined	0 (0)	0 (0)	0 (0)
**EEG-3**	Temporal	29 (85.3%)	27 (93.1%)	22 (91.7%)
	Extratemporal	4 (11.8%)	2 (6.9%)	2 (8.3%)
	Combined	1 (2.9%)	0 (0)	0 (0)
**Overall Chi²**	—	**p < 0.001**	**p < 0.001**	**p < 0.001**

Table 3D. Region of resection by EEG Cluster. Pairwise comparisons. (Bonferroni-corrected significance threshold: p < 0.0167)

**Pairwise Comparisons (Region)**	p-value (Unique cluster patients)	p-value (All ECS 1 patients)	p-value (Unique Cluster Only EC I)
EEG-1 vs EEG-2	<0.001	< 0.001	< 0.001
EEG-1 vs EEG-3	<0.001	0.003	< 0.001
EEG-2 vs EEG-3	P=0.24	0.154	0.186

Table 3E. Outcome by EEG cluster. Unique cluster patients only.

**Outcome**	**EEG-1**<br>**N (%)**	**EEG-2**<br>**N (%)**	**EEG-3**<br>**N (%)**
**EC-1**	12 (%)	31	24
**Not EC-1**	4 (%)	10	10
Total	16 (100)	41 (100)	34 (100)
**Overall chi2**	**p=0.878**		

Table 3F. Outcome by EEG clusters. Unique cluster patients only. Pairwise comparisons.

**Pairwise Comparisons (Outcome)**	p-value (Unique cluster patients)
EEG-1 vs EEG-2	1
EEG-1 vs EEG-3	1
EEG-2 vs EEG-3	0.624

Supplementary Material 4

Table 4A. Side of Resection by Semiology Cluster (Full Cohort and Unique Cluster Subgroup)

Chi-square test for association

**Semiology Cluster**	**Side of Resection**	**A. Unique Cluster Only**	**B. Full Cohort EC 1 (N =103)(%)**	**C. Unique Cluster Only EC 1 (N =64)(%)**
**SEM-1**	Right	1 (25)	2 (20.0)	1 (25.0)
	Left	3 (75)	8 (80.0)	3 (75.0)
**SEM-2**	Right	14 (29.8)	13 (26.0)	9 (27.7)
	Left	33 (70.2)	37 (74.0)	24 (72.7)
**SEM-3**	Right	19 (55.9)	21 (48.8)	16 (59.3)
	Left	14 (41.2)	22 (51.2)	11 (40.7)
	Bilateral	1 (2,9)	0(0)	0(0)
**Overall Chi^2**		P=0.088	**P=0.041**	**P=0.035**

Table 4B. Side of Resection by Semiology Cluster, pairwise comparisons. (Bonferroni-corrected significance threshold: $p < 0.0167$)

**Pairwise Comparisons (Side)**	p-value (Unique cluster only)	p-value (All ECS 1 patients)	p-value (Unique Cluster Only EC 1)
SEM-1 vs SEM-2	1	1	1
SEM-1 vs SEM-3	0.387	0.158	0.304
SEM-2 vs SEM-3	0.023*	0.023*	**0.012**

Table 4C. Region of Resection by Semiology Cluster (Full Cohort and Unique Cluster Subgroup). Chi-square test for association

Semiology Cluster	Region of Resection	Unique Cluster Only	Full Cohort (N =103)(%)	Unique Cluster Only (N =64)(%)
**SEM-1**	Temporal	3 (75)	8 (80.0)	3 (75.0)
	Extratemporal	1 (25)	2 (20.0)	1 (25.0)
**SEM-2**	Temporal	44 (93.6)	49 (98.0)	33 (100.0)
	Extratemporal	2 (4.3)	1 (2.0)	0 (0)
	Combined	1 (2.1)	0 (0)	0 (0)
**SEM-3**	Temporal	19 (55.9)	31 (72.1)	17 (63.0)
	Extratemporal	15 (44.1)	12 (27.9)	10 (37.0)
**Overall Chi²**		**P=0.001**	**P=0.002**	**P=0.001**

Table 4D. Region of Resection by Semiology Cluster, pairwise comparisons. (Bonferroni-corrected significance threshold: p < 0.0167)

**Pairwise Comparisons (Region)**	p-value (Unique clusters only)	p-value (All ECS 1 patients)	p-value (Unique Cluster Only EC 1)
SEM-1 vs SEM-2	0.226	0.069	0.108
SEM-1 vs SEM-3	0.624	1	1
SEM-2 vs SEM-3	**<0.001**	**<0.001**	**<0.001**

Table 4E. Outcome among SEM clusters - unique cluster patients.

**Outcome**	**SEM-1 N (%)**	**SEM-2 N (%)**	**SEM-3 N (%)**
**EC-1**	4 (100.0)	33 (70.2)	27 (79.4)
**Not EC-1**	0 (0)	14 (29.8)	7 (20.6)
Total	4 (100.0)	47 (100)	34 (100)
**Overall chi2**	**p=0.321**		

Table 4F. Outcome within SEM clusters, pairwise comparisons. Bonferroni-corrected significance threshold: $p < 0.0167$

**Pairwise Comparisons (Outcome)**	p-value (Unique clusters only)
SEM-1 vs SEM-2	P= 0.565
SEM-1 vs SEM-3	P=1
SEM-2 vs SEM-3	P=0.351

Supplementary Material 5

Table 5. Distribution of seizure types and key semiological features across three semiology clusters.

Values are presented as n (%). Seizure type comparison based on χ^2 test (3×4 table). Figure-of-four sign analyzed using Fisher's exact test (*). Oroalimentary automatisms analyzed using χ^2 test (3×2 table). Effect sizes are reported as Cramér's V

Variable	Cluster 1 (n=18)	Cluster 2 (n=111)	Cluster 3 (n=91)	p-value	Effect size
FBTCS	18 (100%)	7 (6.3%)	29 (31.9%)	<0.001	Cramér's V ≈ 0.65
FIC	0 (0%)	78 (70.3%)	17 (18.7%)		
FPS	0 (0%)	10 (9.0%)	34 (37.4%)		
Unknown consciousness	0 (0%)	16 (14.4%)	11 (12.1%)		
Figure-of-four sign	18 (100%)	3 (2.7%)	3 (3.3%)	<0.001*	Cramér's V ≈ 0.60
Oroalimentary automatisms	11 (61.1%)	81 (73.0%)	22 (24.2%)	<0.001	Cramér's V ≈ 0.38

FTBS: focal to bilateral tonic-clonic seizures, FIC: focal impaired consciousness (seizures), FPS: focal preserved consciousness (seizures).

Most common semiology features

The most common initial semiology feature in the whole dataset was epigastric aura, reported in 41 seizures, followed by motor or behavioral arrest as the second most common initial feature (onset feature in 30 seizures) and oroalimentary automatisms (onset feature in 23 seizures). When examining epigastric aura as an isolated feature, it was more common in the SEM-3 cluster (23 seizures; 25.3%) than in SEM-2 (17 seizures; 15.3%). When epigastric aura was later during the seizure, followed by automatisms (any kind), the distribution differed; 28 seizures presented epigastric aura followed by automatisms, and 17 of them (60.8%) belonged to cluster SEM-2, while ten (35.7%) belonged to cluster SEM-3. Among the 17 seizures of cluster SEM-2 with this combination, 88.2% were FIC. All thirteen seizures with epigastric aura not followed by automatisms were classified in cluster SEM-3 and more specifically in the subcluster 3a. Nine of these seizures were FPCS. Almost all 27 patients with epigastric aura were patients with temporal lobe epilepsy (25 of 27; 92.6%). The two extratemporal patients had surgery at the insula and frontal area, respectively. The patient with insula resection presented epigastric aura not followed by automatisms, while automatisms (vocal, facial or oroalimentary) occurred after epigastric aura in seizures of the patient with frontal lobe resection. The two extratemporal patients did not belong to semiology cluster 2.

Supplementary Material 6

Potential role of clusters in outcome

To further explore the role of EEG and semiology clusters in predicting surgical outcomes, the dataset was divided into three surgical categories based on resection site: A) Left temporal, B) Right temporal, and C) Extratemporal. For each category, we calculated the proportion of ECS I patients in each cluster and compared it to those not in the cluster.

<u>**For EEG Clusters**</u>

For EEG clusters, the results are presented in *Table 4*. Among patients undergoing left temporal resection, those classified into EEG-3 Cluster had significantly lower rates of ECS I outcome compared to patients not in EEG-3 Cluster (33.3% vs 84.3%, p=0.015). Among patients undergoing extratemporal resection, those classified into EEG-2 Cluster had significantly lower rates of ECS I outcome compared to patients not in Cluster EEG-2 (0% vs 65.0%, p=0.031). For the remaining comparisons, no statistically significant differences were observed between patients belonging to a specific EEG cluster and those not belonging to that cluster.

Table 6A. Comparing proportions of ECS 1 outcome for patients belonging to an EEG cluster vs when not belonging to this cluster. *No observations (right temporal resections) in Cluster EEG-2. **No observations (right temporal resections) not belonging to cluster EEG-3 (right temporal resections in cluster EEG-1 belong at cluster EEG-3 as well, due to overlap). ***one-sided 97.5% CI.

Surgical Category	ECS 1%, In Cluster with 95% CI	ECS 1%, Outside Cluster with 95% CI	p-value
**Left Temporal**	**In Cluster EEG-1**	**Not in EEG-1**	0.261
	92.3%, (64.0-99.8)	75.0%, (59.7-86.8)	
	**In Cluster EEG-2**	**Not in EEG-2**	0.38
	80%, (67.0-89.6)	50%, (1.3-98.7)	
	**In Cluster EEG-3**	**Not in EEG-3**	**0.015**
	33.3%, (4.3-77.7)	84.3%, (71.4-93.0)	
**Right Temporal**	**In Cluster EEG-1**	**Not in EEG-1**	0.591
	60.0%, (14.7-94.7)	75.9%, (56.5-89.7)	
	**In Cluster EEG-2**	**Not in EEG-2**	-
	-*	73.5%, (55.6-87.1)	
	**In Cluster EEG-3**	**Not in EEG-3**	-
	73.5%, (55.6-87.1)	**-****	
**Extratemporal**	**In Cluster EEG-1**	**Not in EEG-1**	0.357
	61.1%, (35.7-82.7)	33.3%, (4.3-77.7)	
	**In Cluster EEG-2**	**Not in EEG-2**	**0.031**
	0(%), (0-60.2***)	65.0%, (40.8-84.6)	
	**In Cluster EEG-3**	**Not in EEG-3**	0.63
	40.0%, (5.3-85.3)	57.9%, (33.5-79.7)	

For Semiology Clusters:

In the analysis of semiology clusters, no statistically significant associations were identified between cluster membership and achieving an ECS I outcome across any of the three surgical categories. Although certain comparisons suggested a trend toward outcome differences—such as a lower rate of ECS I outcome for patients in SEM-1 Cluster among right temporal resections (33.3% vs. 80.0%, *p*=0.145) and for patients in SEM-2 Cluster among extratemporal resections (20.0% vs. 63.2%, *p*=0.142)—these findings did not reach statistical significance. Detailed results are presented in *Table 5*.

Table 6B. Comparing proportions of ECS 1 outcome for patients belonging to a Semiology cluster vs when not belonging to this cluster.

Surgical Category	ECS 1 %, In Cluster$_{(x)}$ (95% CI)	ECS 1%, Outside Cluster$_{(x)}$ (95% CI)	p-value
**Left Temporal**	**In SEM-1**	**Not in SEM-1**	1
	87.5%, (47.3-99.7)	77.5%, (63.4-88.2)	
	**In SEM-2**	**Not in SEM- 2**	1
	78.3%, (63.6-89.1)	81.8%, (48.2-97.7)	
	**In SEM-3**	**Not in SEM-3**	0.736
	75.0%, (50.9-91.3)	81.1%, (64.8-92.0)	
**Right Temporal**	**In SEM-1**	**Not in SEM-1**	0.145
	33.3%, (0.8-90.6)	80.0%, (59.3-93.2)	
	**In SEM- 2**	**Not in SEM- 2**	0.107
	61.9%, (38.4-81.9)	92.3%, (64.0-99.8)	
	**In SEM- 3**	**Not in SEM- 3**	0.435
	80.0%, (56.3-94.3)	64.3%, (35.1-82.7)	
**Extratemporal**	**In SEM-1**	**Not in SEM-1**	0.63
	40.0%, (5.3-85.3)	57.9%, (33.5-79.7)	
	**In SEM-2**	**Not in SEM-2**	0.142
	20.0%, (0.5-71.6)	63.2%, (38.4-83.7)	
	**In SEM- 3**	**Not in SEM- 3**	0.576
	57.1, (34.0-78.2)	33.3, (0.8-90.6)	

When examining the outcome in the entire dataset (regardless cluster classification), we found that patients with extratemporal resection had 65% lower odds of having ECS I at 12 months post-surgery, compared to patients with temporal resection: OR=0.35, p=0.031, [95%CI: 0.14 - 0.91]. After adjusting for sex and age at surgery time, the odds ratio remained significant: OR: 0.31, p=0.022, [95%CI: 0.11-0.85].

When comparing outcomes between patients in multiple EEG clusters and those in a single cluster, 36% of the former did not achieve ECS I, compared to 26.4% in the latter, though this difference was not statistically significant (p = 0.232). We also compared the proportion of ECS I patients with left or right temporal resections in single EEG clusters (EEG-2 or EEG-3; 77.9%) to those in multiple clusters (EEG-1 & EEG-2, EEG-1 & EEG-3, or EEG-2 & EEG-3;

72.7%), finding no significant difference ($p = 0.618$). Similarly, a trend showed that 38.7% of patients in multiple semiology clusters did not achieve ECS I, compared to 24.7% in a single cluster, though this difference was not significant ($p = 0.14$).

Supplementary Material 7A. Accuracy of individual semiology features for identifying temporal lobe epilepsy

Table 7A. Accuracy of individual semiology features or combinations for identifying temporal lobe epilepsy.

Semiology Feature	Sensitivity (95% CI)	Specificity (95% CI)	Accuracy (95% CI)	Diagnostic OR (95% CI)
**Epigastric aura**	33.3% (22.7-45.4%)	95.2% (76.2-99.9%)	47.3% (36.9-57.9)	10.0 (1.27-79.0)
**Manual, distal automatisms**	52.1% (39.9-64.1%)	58.3% (36.6-77.9)	53.7 (43.2-64.0)	1.52 (0.60-3.88)
**Oroalimentary automatisms**	67.6% (55.7-78.0)	41.7% (22.1-63.4)	61.2% (50.9-70.9)	1.49 (0.58-3.83)
**Any automatism (manual, oroalimentary, vocal, verbal)**	82.7% (72.2-90.4)	25.0% (9.8-46.7)	68.7% (58.6-77.6)	1.59 (0.53-4.78)
**Combination of epigastric aura & any automatism**	26.8% (16.9-38.6)	95.2% (76.2-99.9)	42.4% (32.2-53.1)	7.31 (0.92-58.3)
**Ictal dystonia**	27.1% (17.2-39.1)	58.3% (36.6-77.9)	35.1% (25.5-45.6)	0.52 (0.20-1.37)
**Combination of ictal dystonia & manual, distal automatisms**	21.4% (12.5-32.8)	75.0% (53.3-90.2)	35.1% (25.5-45.6)	0.82 (0.28-2.42)

OR: odds ratio

Supplementary Material 7B – Lateralization value of semiology features (only temporal lobe resections included)

Patients who presented the examined feature in both sides across their multiple seizures but had one-sided resection were excluded from this analysis.

Table 7B. Lateralization value of individual semiology features in temporal lobe resections. 1. Only FBTC included – in cases of forced head version more than once during the seizure, the head version just prior to the bilateral tonic-clonic jerks was included. 2. Head orientation (not forced version) occurring as one of the first three semiology signs was included. *one-sided, 97.5% CI.

Lateralizing Feature	Lateralization Hypothesis	% correctly identified cases (all outcomes), 95%CI	% correctly identified cases (Engel Class I), 95%CI
Manual, distal automatisms	Ipsilateral	86.8%, (71.9%-95.6%)	86.7% (69.3-96.2)
Ictal dystonia	Contralateral	100%, (88.1-100%*)	100%, (82.4-100*)
Ictal/postictal aphasia	Dominant Hemisphere	93.5%, (78.6-99.2%)	95.5%, (77.2-99.9)
Head version prior to secondary generalization[1]	Contralateral	89.3%, (71.8-97.7%)	86.4%, (65.1-97.1)
Early head orientation[2]	Ipsilateral	93.3%, (95%CI: 68.1-99.8%)	100%, (69.2-100*)
Last clonic jerks	Ipsilateral	87.7%, (95%CI: 61.7-98.5%)	84.6% (54.6-98.1)
Postictal nose wiping	Ipsilateral	92.3%, (74.9-99.1)	90.1% (69.6-98.8%)
Partial responsiveness & automatisms	Non-dominant hemisphere	41.7%, (15.2-72.3%)	50%, (15.7-84.3%
