# Supplementary material for: Unsupervised clustering of electroclinical features in temporal lobe epilepsy: A data‐driven approach
Source: Epileptic Disord. 2026 May 19;28(4):1236–51. doi: 10.1002/epd2.70283 (PMC13499273; doi:10.1002/epd2.70283)
Supplement: Supplementary file 2 [file EPD2-28-1236-s002.docx]

**TEST YOURSELF**

**Answers**

**1. C**

**2. C**

**3. C**
